# Nerve and Arterial Supply Pattern of the Popliteus Muscle and Clinical Implications

**DOI:** 10.1155/2022/6980471

**Published:** 2022-01-10

**Authors:** Anna Jeon, Ye-Gyung Kim, Youngjoo Sohn, Je-Hun Lee

**Affiliations:** ^1^Department of Anatomy, College of Medicine, Ewha Womans University, Seoul, Republic of Korea; ^2^Department of Anatomy, College of Medicine, Chung-Ang University, Seoul, Republic of Korea; ^3^Department of Anatomy, College of Korean Medicine, Kyung Hee University, Seoul, Republic of Korea; ^4^Korea Institute for Applied Anatomy, College of Sports Science, Korea National Sport University, Seoul, Republic of Korea

## Abstract

**Introduction:**

The aim of this study was to investigate the nerve and artery supply and the tibial attachment of the popliteus muscle using anatomical methods.

**Methods:**

Forty-four nonembalmed and embalmed extremities were dissected for this study. To measure the attachment area of the popliteus, the most prominent points of the medial epicondyle of the femur and the medial malleolus of the tibia were identified before dissection. A line connecting these two prominent points was used as the reference line, with the most prominent point of the medial epicondyle of the femur as the starting point. This study also investigated the area where the popliteus attaches to the bone and the points where nerves and arteries enter the popliteus muscle when it is divided into three equal parts in the coronal plane.

**Results:**

The mean length of the reference line was 34.6 ± 2.1 cm. The origin of the popliteus was found to be at a distance of 16.6% to 35.2% on the tibial bone from the proximal region. The popliteus was innervated by only the tibial nerve in 90% of the cases and by the tibial and the sciatic nerves in the remaining 10% of the cases. The inferior medial genicular artery and the posterior tibial artery supplied blood to the popliteus in 90% and 65% of the cases, respectively. When the popliteus muscle was divided into three equal parts in the coronal plane, the nerve and the artery were found to enter the muscle belly in zones II and III and zones I and II in 92% and 98% of the specimens, respectively. *Discussion*. The anatomical investigation of the popliteus in this study will help identify patients with clinically relevant syndromes.

## 1. Introduction

The popliteus muscle (PM) is a small muscle that acts as a major posterolateral stabilizer of the knee joint, rotating the tibia medially under the femur under non-weight-bearing conditions [1, 2]. As the PM acts as an important factor in the movement and injury of the knee joint, anatomical studies have been conducted with a focus on the femoral attachment of the muscle [1, 3–8]. A study using magnetic resonance imaging analysis showed that the PM sulcus depth affected the rotational alignment with tendinitis [9]. The PM sulcus is also considered to be on the femoral side of the PM muscle. Although a morphological classification of the PM was recently performed [10], no studies on nerve innervations and blood supply were found.

Anatomy textbooks state that the tibial nerve innervates the PM [11, 12]; there is also study showing that the tibial nerve innervates [2]. However, it is difficult to obtain information about the nerve supply and distribution from the tibial nerve to the PM in the popliteal region. Moreover, this cadaveric study was conducted as there is little information about the arterial supply.

Muscular spasticity is common in upper motor neuron syndrome. Injection treatment is applied as PM spasticity has been confirmed in many patients with in-toeing [2]. One of the treatment methods, botulinum toxin, is known to have a long-lasting effect when injected into a site where the neuromuscular junction is dense. It is also effective when injected near the motor entry point where the nerve enters the muscle belly [13]. Thus, a suitable injection site is thought to be the tibial region because the muscle belly is the upper portion on the tibial area on the posterior aspect. In this study, we speculated about the injection site of the PM based on the above reasoning. An alternative method is the accurate palpation of the PM, which is necessary for posture correction therapy. However, there is insufficient data on how to accurately palpate the PM.

Thus, the aim of this study was to investigate the nerve and artery supply of the tibial attachment of the PM using anatomical methods.

## 2. Materials and Methods

Twenty-three adult cadavers (13 males and 10 females, age range 45–95 years) donated to the medical university were dissected for this study. Two limbs showing evidence of prior surgery or injury around the popliteal region were excluded.

**Figure 1:**
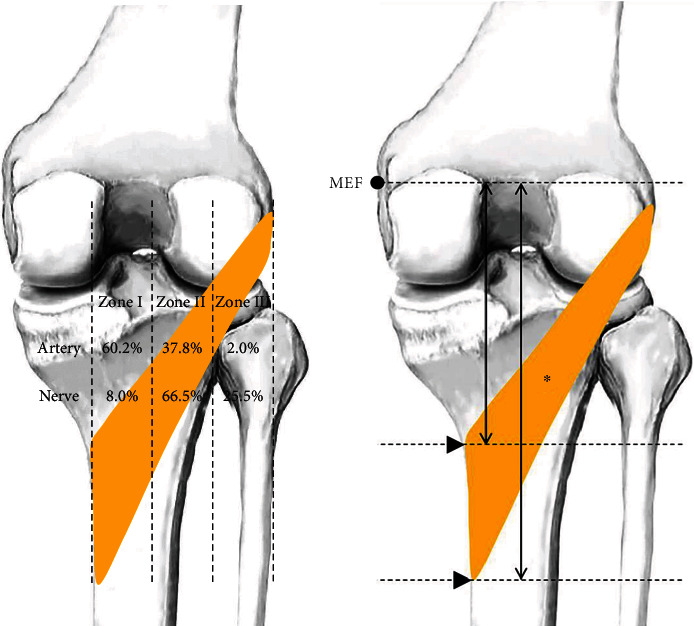
Illustration of the reference lines used to measure the blood supply and innervation of the popliteus muscle (asterisk) and its area of attachment. (a) The entry point of the nerve with the artery when the popliteus was divided into three equal parts in the coronal plane. (b) The attachment area of the popliteus muscle.

For the dissection procedure, the skin was removed to expose the fatty layer and superficial fascia, and then, the heads of the gastrocnemius were carefully cut to identify the PM and neurovascular structures surrounding the PM. Further careful dissection was performed to identify the nerve branches around the PM, and the artery entering the PM was traced. After dissection of the neurovascular structures around the PM, this study investigated the tibial attachment area and the entry points of the nerve and the artery when the PM muscle was divided into three equal parts in the coronal plane (Figure 1(a)). To measure the point of attachment of the PM in this study, the medial border of the tibia and the prominent point of the lateral epicondyle of the femur were divided into three parts at equal intervals (Figure 1(a)).

To measure certain variables, the most prominent points of the medial epicondyle of the femur (MEF) and the medial malleolus of the tibia MMT were identified before dissection. A line connecting the MEF and the MMT was used as the reference line, with the MEF as the starting point (Figure 1(b)). The measurement variables were as follows:

1. The reference line between the MEF and MMT
2. The proximal attachment point of the PM from the MEF
3. The distal attachment point of the PM from the MEF

A single observer obtained all measurements using a measuring tape and digital calipers (resolution 0.01 mm, CD-20PSX, Mitutoyo, Japan). The data were analyzed using SPSS version 23.0 (IBM SPSS Inc., Chicago, IL, USA). The present study was conducted in accordance with the principles of the Declaration of Helsinki. This study was approved by the Institutional Review Board of Korea National Sport University (IRB No. 20210722-115).

## 3. Results

The mean length of the reference line was 34.6 ± 2.1 cm. The attachment point of the PM was located at a distance of 16.6% to 35.2% along the tibial bone from the proximal region (Table 1). No significant differences were found in the reference line or between the right and left legs between males and females (*p* ≥ 0.05).

**Figure 2:**
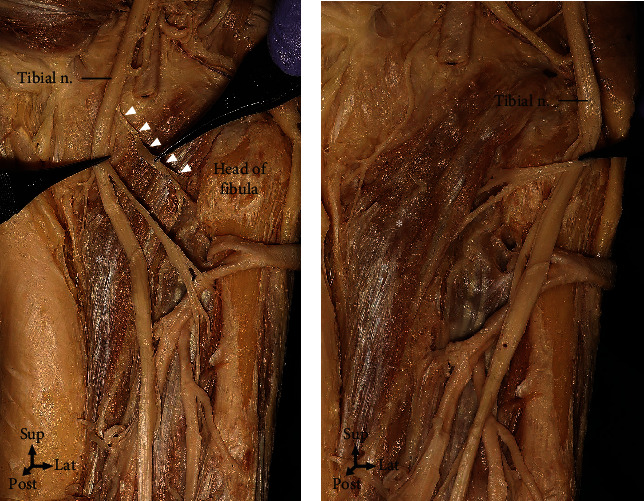
The popliteus (asterisk) was innervated by branches extending from the tibial nerve (arrowheads) in 90% of the cases. (a) The branches of the tibial nerve rotated at the lower border of the popliteus and entered deep into the muscles. (b) An anatomical photograph showing the tibial nerve branch entering deep into the popliteus in detail.

**Figure 3:**
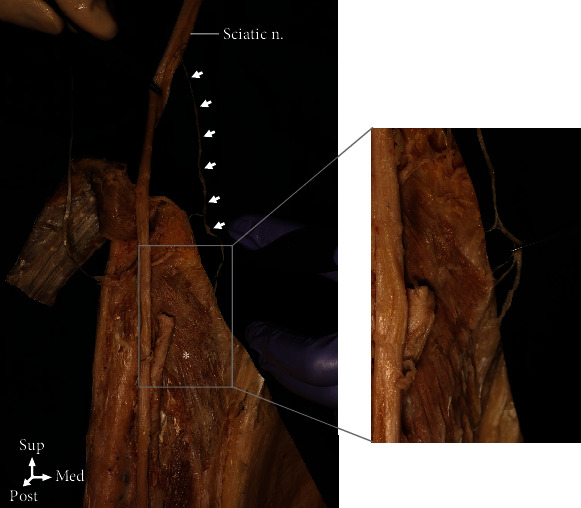
The popliteus (asterisk) was occasionally innervated by branches extending from the sciatic nerve (arrows) in 10% of the cases.

In 90% of the cases, the PM was innervated by the tibial nerve only, whereas the PM was innervated by the tibial nerve and the sciatic nerve in the remaining 10% of the cases (Figures 2 and 3). When the PM was divided into three equal parts in the coronal plane for the nerve division, zones II and III were distributed at 66.5% and 25.5%, respectively. The artery was distributed at 60.2%, 37.8%, and 2.0% in zones I, II, and III, respectively (Figure 1(a)).

**Figure 4:**
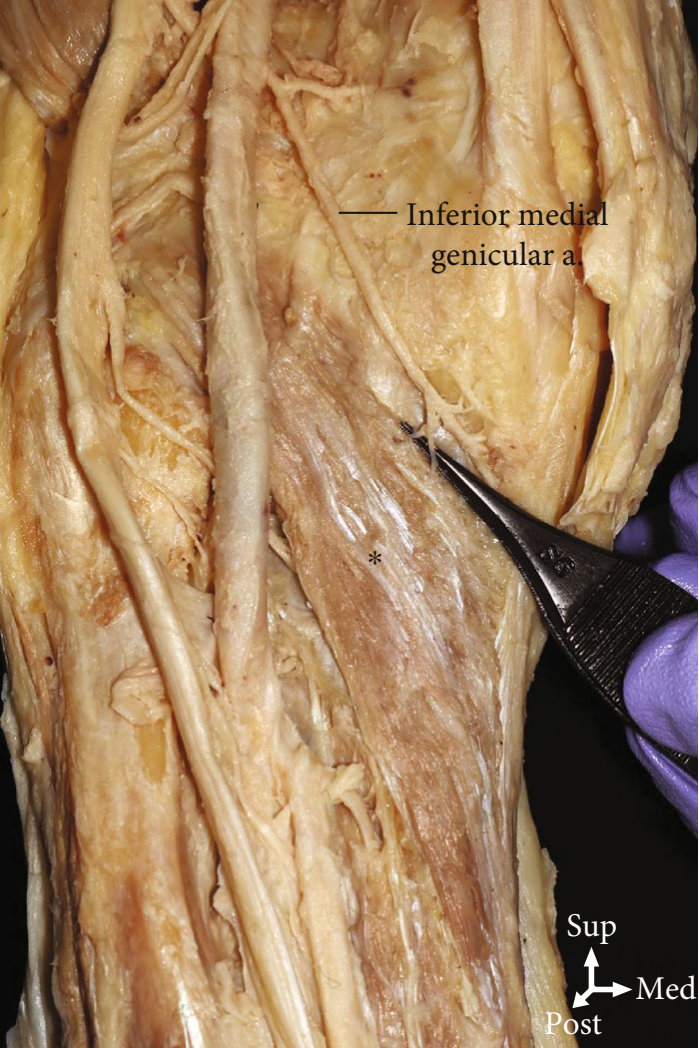
The arterial supply of the popliteus (asterisk) was derived from the inferior medial genicular arteries in 90.0% of the cases.

**Figure 5:**
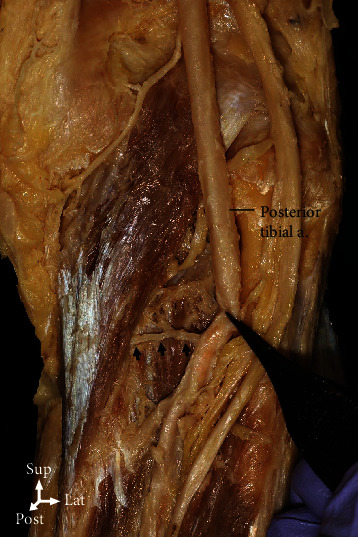
The posterior tibial artery (arrows) was involved in the blood supply to the popliteus (asterisk) in 65% of the cases.

**Figure 6:**
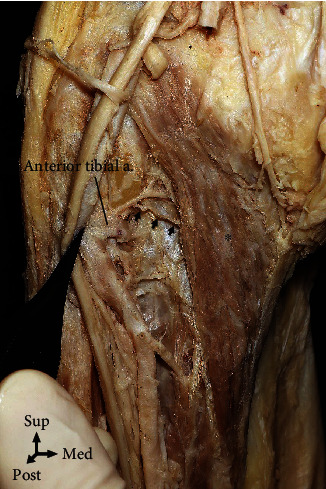
The blood supply of the popliteus (asterisk) was by the anterior tibial arteries (arrows) in 35.0% of the cases.

**Figure 7:**
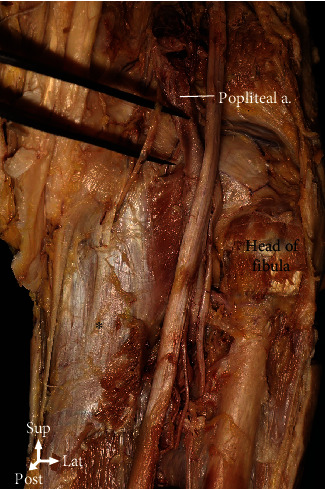
The popliteal artery additionally contributed to the popliteus (asterisk) in 5% of the cases.

Upon examination of the arteries that supplied blood to the PM, one to three arteries were found to supply blood. In this investigation, when the sum of the arterial frequencies was calculated, the inferior medial genicular artery was involved in supplying blood to the PM in 90.0% of the cases (Figure 4). In 65% of the cases, the posterior tibial artery was involved in supplying blood to the PM (Figure 5). The blood supply to the PM was by the anterior tibial artery in 35.0% of the cases and by the popliteal artery in 5.0% of the cases (Figures 6 and 7, Table 2).

## 4. Discussion

The PM is a major stabilizer of the posterolateral knee region, and overactivity of the PM might lead to friction between the PM tendon, lateral femoral condyle, and the lateral meniscus. PM shortness is a common cause of lateral knee pain [14, 15]. Immobilization could tighten the PM, resulting in strain following a twisting strike during weight-bearing. Identifying a PM lesion is challenging because the muscle is deeply situated and its pain is difficult to differentiate from that of the adjacent structures, such as the gastrocnemius, meniscus, and posterior cruciate ligament [16]. One study reported that PM syndrome was caused by an isolated PM due to compression of the neurovascular bundle. In this case, the patient felt calf pain and numbness in the posterolateral aspect of the calf and sole [17]. Because the PM and popliteal artery are adjacent to each other, the condition of this muscle affects the blood circulation of the popliteal artery. To date, few anatomical studies have been conducted on the blood supply of the PM. In this study, the blood supply of the PM was often found to be provided by the inferior medial genicular artery (Figure 4). Blood supply is important for muscle recovery. Thus, adequate blood supply is necessary for the recovery of the PM as the legs are mostly in standing and working positions. In addition, anatomical studies on the arteries supplying blood to the PM will help increase the success rate of surgery by providing information on the supply of nutrients to the PM during surgery.

The number and location of the motor entry points of the PM are also clinically interesting for effective muscle spasticity treatment. Earlier studies [2] reported an average of 2.2 motor entry points (range, 1–3) with the tibial branch as the origin of the innervating nerve for the PM and the nerve branch entering the PM on its superficial surface ~1 cm distal to its superior border. In this study, the number of motor entry points ranged from 1 to 4. The most common cases had two motor entry points (47.6%), followed by one (33.3%) and three motor entry points (14.3%). Four motor entry points were found in 4.8% of the cases. In all cases in this study, the innervating nerve for the PM did not originate from the tibial nerve. In 10% of the cases, the PM was innervated by the tibial and the sciatic nerves (Figure 3), and the nerve entered the inferior border of the PM in almost all the cases (Figure 2). In this study, as 66.5% of the motor entry points were distributed in zone II, zone II was considered to be the most effective injection point (Figure 1).

Next, we surveyed the results of previous studies examining the shape of the PM and the surrounding structures to which it is connected [1, 16, 17]. The PM was shown to have a triangular shape [2] and is attached to surrounding ligaments and meniscus [1]. A recent study investigated the shape of PM tendon and the surrounding structures it is connected to during its insertion to bone [10]. Another study examined the shape related to the lateral collateral ligament at the point where the PM is inserted [18]. In this study, we focused on how the origin site attaches to the bone and the nerve and the artery supplying the PM rather than the surrounding structures.

One method uses manual therapy for pain caused by PM shortness. A point situated between 16.6% and 35.2% of the distance from the MEF on the tibial bone was found to be suitable for palpation of the PM (Table 1). Knowledge of the location of the origin of attachment of the PM to the tibia will be helpful in the treatment of muscle relaxation by applying appropriate pressure through manual therapy. Previous research [2] showed the shape of the PM but from a posterior view. There are structures superficial to the PM, such as the gastrocnemius, nerves, and vessels. Therefore, it is recommended to approach the PM by touching the bone at the medial border of the tibia. The results of this study suggest that setting the area between 20 and 30% along the medial border of the tibia would be optimal for manual therapy for tight PM (Table 1).

## 5. Conclusions

The present study showed that the tibial attachment area, nerve innervations, and blood supply of the PM could be considered constant anatomical characteristics. We propose that manual treatment targeting the PM be located 20–30% from the MEF along the tibial bone. In 90% of the specimens, the PM was innervated by the tibial nerve and received blood from the inferior medial genicular artery. When the PM was divided into three equal parts in the coronal plane, the nerve was found to enter the muscle belly in zones II and III in 92.0% of specimens, whereas the artery entered the muscle belly in zones I and II in 98.0% of specimens (Figure 1). This finding will help understand the anatomy of the PM and its environment and treatment of syndromes involving the PM.

## Figures and Tables

**Table 1:** Total length of the reference line and attachment point of the popliteus on the bone.

Total length	Proximal point	Distal point
34.6 ± 2.1	5.8 ± 1.3 (16.6 ± 3.6%)	12.1 ± 1.3 (35.2 ± 3.9%)

Unit: cm.

**Table 2:** Origin of the artery supplying blood to the popliteus muscle.

Artery	%
Anterior tibial a. and inferior medial genicular a.	25
Inferior medial genicular a. and posterior tibial a.	20
Inferior medial genicular a.	25
Popliteal a. and inferior medial genicular a. and posterior tibial a.	5
Posterior tibial a. and anterior tibial a.	5
Anterior tibial a.	5
Posterior tibial a.	15

## Data Availability

The underlying data supporting the results of our study can be found in the manuscript.
